# Old Age and the Modifications in the Course of the Ordinary Diseases When They Attack the Aged

**Published:** 1899-11

**Authors:** Marvin E. Nuckols

**Affiliations:** Lecturer on Medical Jurisprudence and Assistant Demonstrator of Chemistry, University College of Medicine, Richmond, Va.


### Old Age and the Modifications in the Course of the Ordinary Diseases When They Attack the Aged.

BY MARVIN E. NUCKOLS, M. D.,
Lecturer on Medical Jurisprudence and Assistant Demonstrator of Chemistry, University College of Medicine, Richmond, Va.

In considering the subject of old age the question arises, is it a disease, as the older and, in fact, some of the more recent writers would have us believe, or is it a physiological condition? Before we answer this question we must determine what the normal physiological state is. If we take the functions of a healthy adult as a standard and compare them with those of the old man, or even the child, it is easy to note the most striking difference, not only in the physiological functions, but also in the anatomical structure of the organs. Since we do not regard childhood (a period in which the functions are nearly as different from those of the adult as are the functions in old age) as a disease, why should we call old age a disease?

Old age may be defined as a modification of the normal condition (taking the adult as normal), in which all the functions of the organs are retarded, but acting in perfect harmony, and it is, therefore, not a disease, for disease implies a rupture of relations or lack of harmony between the various organs; consequently we can only look upon old age as a normal phase of life, a period of slow and gradual retrogression towards death and as true a physiological condition as childhood or adult life. To make this clearer and more conclusive, let us observe for awhile the living being, following it through life. The essential characters of living matter are instability and power of attraction, or the power by which the cells are nourished or new ones produced. This power resides in and is an inherent property of the protoplasm of the cells, and by it the process of integration and repair is explained, and, by its diminution, disintegration and destruction; so it can be readily seen that this characteristic plays an important part in the maintenance of life.

A natural conclusion from the above would be that living matter ought never to die, but should be immortal. It possesses a strong power of attraction, its instability enables it to accommodate itself to any environment, and its surroundings furnish all the material necessary for its maintenance. But cells in the course of their existence become differentiated; that is, they are changed from the primitive type; they attain a higher degree of perfection; their functions are exhalted; they become specialized, and, in doing this, they lose some of their power of attraction; their resistance is lessened; in other words, what they give in quality they lose in quantity. But, fortunately, all cells are not highly differentiated, and all do not reach their maturity at once. If they did our lives might be shorter than they are. The nerve cell is probably the most highly differentiated and is the first to wear out, other things being equal; while the connective-tissue cell is the least differentiated, and as a result enjoys a long and happy existence. This may be more clearly shown by an example. Suppose nervous tissue, or any other highly-developed tissue, is worn out through use, or has accidentally been rendered incapable of performing its functions. It cannot replace itself, but is replaced by connective tissue, thus explaining the extent of sclerosis in old age. In connection with this, I do not think it will be out of place to say something of the rôle which connective tissue plays in the economy. It is the most widely distributed of all the tissues, acting as a support and protection for tissues more highly organized. It is of all the tissues the most capable of regeneration, acting the principal part in healing all wounds and injuries. When more highly developed tissue is worn out or destroyed it takes its place, or it may be converted into cells identical with those destroyed, and thus replace them. In this case the connective-tissue cells seem, as it were, to forget themselves and assume the character and functions of their neighbors. Prolonged excitement due to hyperemia, hyperactivity of function and circulation of tox'c blood will also cause its development. This process is constantly going on in later life, and we should not be surprised to find sclerosis of the organs of the body. When the noble cells, or highly differentiated cells, cannot be replaced in sufficient quantity, and connective tissue begins to fill in the gaps, the functions of the organs are interfered with and senescence begins. It may be asked, why old age begins at different periods in different individuals. This is explained by difference in habits, temperament and diathesis, all of which affect the resistance of the cells. A person may inherit a strumous diathesis, in which the cells have little vitality from the beginning, little power of attraction and little resistance, and, as a result, are prone from the beginning to early degeneration and death. It is easy to understand why this person becomes old sooner than one who inherits no diathesis and lives a temperate life.

Various theories have been advanced from time to time as to the causes of senility, but I shall mention only two to show how different they are from the present teaching:

The first is that the cause resides in the respiratory organs. The lungs perform their functions imperfectly, resulting in defective hemastosis; the blood is not sufficiently oxygenated, and, as it is the carrier of nutrition, the organs and tissues suffer as a consequence. The second is that sclerosis is produced by arteritis, and this, in turn, by vitiated blood. By deductive reasoning, we come back to vitiated blood as the primary cause of senility, but no cause is given for vitiated blood.

The great objection to these theories is that they take effects for causes. All these conditions are the results of senility, as I have shown when speaking of the evolution and specialization of cells.

Now that it has been shown what old age really is, and how it is brought about, I will say something of it in its relation to the ordinary diseases.

The older writers tell us that there is a defective reaction to disease, and that the organs seem to become independent of one another and to suffer separately; in other words, the condition of a particular organ is not echoed by the economy as a whole, as in adult life. This is to a certain extent true, but when we study the conditions more closely we find that it is more apparent than real. Consider the temperature in febrile conditions in the aged. To the touch, or if taken in the axilla, it is very much lower than that of an adult under the same condition. The subcutaneous fat has disappeared and the circulation in the skin is poor, and as a consequence the skin and extremities may feel cold, but if the temperature is taken in the rectum it corresponds very nearly to that of the adult. That there is reaction in the aged is also shown by increased combustion in febrile conditions, manifested by an increase in the solids of urine; but reaction, while marked, is somewhat less than in adult life or childhood. The reason for this is that the functions are retarded. The organs which express and feel are slower to act. The whole condition is one of lowered vitality. The pulse is usually below seventy and is often intermittent, showing that the heart, like all the organs, is wearing out and needs rest. A pulse of 80 to 85 usually indicates some pathological condition, and, when noticed, necessitates a thorough examination in order to discover its cause, while a pulse of 120, if it lasts any length of time, is almost certainly fatal.

The respiration is always increased, even when the lungs are not involved (but it is exceedingly rare to find an old person whose lungs have not undergone some change, either fibroid or emphysematous, or both), due to nature's endeavor, that hemastosis may go on properly.

The eruptive diseases are rare in the aged, because most old people have had them; still those who have never had them are not immune. The symptoms are never characteristic, the eruption is almost absent, complications are more frequent, the heart, lungs and kidneys being especially apt to be involved.

Typhoid fever is not rare in the aged. The local symptoms are generally mild. The fever is of a low type; toxemia and depression are always marked, and convalescence is tardy and relapses frequent.

Bronchitis in the aged is very frequent, both on account of the conditions already present in the lungs—emphysema, sclerosis and dilatation of the bronchi—and the natural susceptibility of the aged to cold. It is apt to become chronic, especially in those who are gouty. It also shows a marked tendency to extend to the smaller bronchial tubes, thus jeopardizing life.

Pneumonia is probably the most frequent acute disease of old age, and kills more old people than any other disease. It develops in an abnormal manner, and is very insidious in its onset. The objective symptoms are not characteristic; in fact, the patient may present the symptoms of meningitis or no symptoms at all, and may even be going about when the physical signs of pneumonia are present. Therefore, when called to see the aged in winter, even if there are no outward manifestations of pneumonia, it is advisable to examine the lungs. The symptoms, when present, are slight, low fever, little or no pain, some dyspnea and marked cyanosis. Tuberculosis, once said not to exist in old age, is sometimes present, being usually of the fibroid tvpe. In it there is little or no cough or fever, no night sweats—simply slow and progressive emaciation, with the physical signs.

Acute Bright's disease probably does not exist in the aged. The kidneys are always atrophied and degenerated; still the system accommodates itself to the condition, and the patient gets on fairly well until there is some disturbance in the economy; katabolism is increased, and the kidneys, being unable to eliminate the waste material, uremia results. This may be so slight at times, giving rise to only headache and malaise, that we may be thrown off the track unless frequent examinations of the urine are made.

I might go on indefinitely with a description of the diseases occurring in the aged, but, since I have spoken of the principal diseases common to other periods of life as well as old age, I shall conclude with a few remarks as to the care of the aged.

We should especially advise them with reference to diet, fresh air, exercise and clothing. The diet should be simple, but nutritious. The patients should aim to eat simply to repair, and as the functions are much reduced, very little is required to maintain life. They should eat as do children—often and in small quantities. The evening meal should be light and unstimulating. We should caution them especially about overloading the stomach and allowing themselves to become constipated, as this is one of the most frequent exciting causes of apoplexy. Alcoholic liquors, if drunk at all, should be taken with caution.

Fresh air is very essential to the aged; in fact, more so than at any period of life, because, on account of the condition of the lungs, hemastosis is imperfect. It is entirely wrong to keep an old man shut up in a warm room and allow him to breathe and rebreathe the same air. It is a common thing to find an old man, heavily clothed, drawn up by a hot fire, and dreading a breath of fresh air for fear of taking cold. The clothing should be warm, but light, and he should be made to take outdoor exercise every day.

When called upon to treat the aged we should make a thorough examination, especially of the lungs, heart and kidneys, for reactions in the aged are not marked, and some serious condition may exist without any outward manifestation. In all diseases we should test the urine from day to day, and should be ever ready to anticipate and combat the depression, so often seen in the course of diseases in the aged, by the administration of tonics and stimulants.

